# Inflammatory bowel disease-specific findings are common morphological changes in the ileal pouch with ulcerative colitis

**DOI:** 10.1038/s41598-022-24708-2

**Published:** 2022-11-27

**Authors:** Kenichiro Toritani, Hideaki Kimura, Masako Otani, Hironori Fukuoka, Reiko Kunisaki, Jun Watanabe, Atsushi Ishibe, Toshihiro Misumi, Yoshiaki Inayama, Itaru Endo

**Affiliations:** 1 Department of Gastroenterological Surgery, Yokohama City University Graduate School of Medicine, Yokohama, Japan; 2 Inflammatory Bowel Disease Center, Yokohama City University Medical Center, 4-57, Urafune-Cho, Minami-Ku, Yokohama, 232-0024 Japan; 3 Diagnostic Pathology, Yokohama City University Medical Center, Yokohama, Japan; 4 Department of Surgery, Gastroenterological Center, Yokohama City University Medical Center, Yokohama, Japan; 5 Department of Biostatistics, Yokohama City University School of Medicine, Yokohama, Japan

**Keywords:** Inflammation, Gastrointestinal diseases

## Abstract

Why inflammation is common in ileal pouches with ulcerative colitis (UC) is unclear. We therefore clarified the morphological changes in pouches and afferent limbs (AL) of patients with UC and explored the relationship between these findings. We evaluated the morphological findings (histological and endoscopic inflammation as the Pouchitis Disease Activity Index [PDAI] histology subscore [hPDAI] and endoscopy subscore [ePDAI], inflammatory bowel disease [IBD]-specific findings using the IBD score [S_IBD_], colonic metaplasia using the colonic metaplasia score [CMS], and goblet cell [GC] ratio) in the pouch and AL of patients with UC. A total of 261 pouchoscopies were analyzed. The pouch body had a higher hPDAI (*p* < 0.001), S_IBD_ (*p* < 0.001), CMS (*p* < 0.001), GC ratio (*p* < 0.001), and ePDAI (*p* < 0.001) than the AL. The hPDAI was correlated with the S_IBD_ (Spearman’s coefficient r = 0.538; *p* < 0.001), CMS (r = 0.687; *p* < 0.001), and the ePDAI (r = 0.552; *p* < 0.001), but not with GC ratio (r = 0.175; *p* < 0.001) or the pouch usage duration (r = −0.057; *p* = 0.107). The incidence of histological inflammation was higher in specimens showing basal plasmacytosis with severe mononuclear cell infiltration (BP) than in those without BP (odds ratio [OR] 6.790, *p* < 0.001), BP was commonly found with crypt hyperplasia (OR 3.414, *p* < 0.001) and the crypt length correlated with neutrophil infiltration (r = 0.469; *p* < 0.001). Histological inflammation, colonic metaplasia, the GC ratio, endoscopic inflammation, and IBD-specific findings were commonly present in the pouch than in the AL. Histological inflammation occurs with IBD-specific findings and colonic metaplasia, and these signify endoscopic inflammation.

Ulcerative colitis (UC), an inflammatory bowel disease (IBD), has spread worldwide, with an increasing incidence in non-Western countries and remains highly prevalent in Western countries^1^. Even in the era of biologics, total proctocolectomy is still required by 10–30% of patients with UC^2^. Since the invention of the ileal pouch^3^, which recreates a fecal reservoir, many patients have been freed from the ileostomy, which has greatly improved their quality of life (QOL)^4^.

Despite these benefits, approximately half of UC patients who received pouch surgery suffer from late complications, such as inflammation of the pouch (pouchitis)^5^. Interestingly, this inflammation rarely occurs in the ileostomy of patients with UC or in the ileal pouch of patients with familial adenomatous polyposis (FAP)^6^. Therefore, fecal stasis in the pouch body and recurrence of IBD are thought to be related to pouchitis; however, the etiology remains unclear. Clinically, the endoscopic and histological morphology of the afferent limbs (AL) and the pouch body (the proximal pouch [PP] and the distal pouch [DP]) differ in patients with UC, and this difference may help clarify why pouchitis is so common in the UC pouch.

The present study clarified the morphological differences (histological and endoscopic inflammation as the Pouchitis Disease Activity Index histology subscore [hPDAI]^7^ and endoscopy subscore [ePDAI]^7^, inflammatory bowel disease [IBD]-specific findings as the IBD score [S_IBD_]^8^, colonic metaplasia as the colonic metaplasia score [CMS]^9^, and goblet cell [GC] ratio as the ratio of goblet cells among epithelial cells) between AL and the pouch body in patients with UC as well as the relationship among these morphological findings.

## Results

### Morphological difference between AL, PP and DP

**Figure 1:**
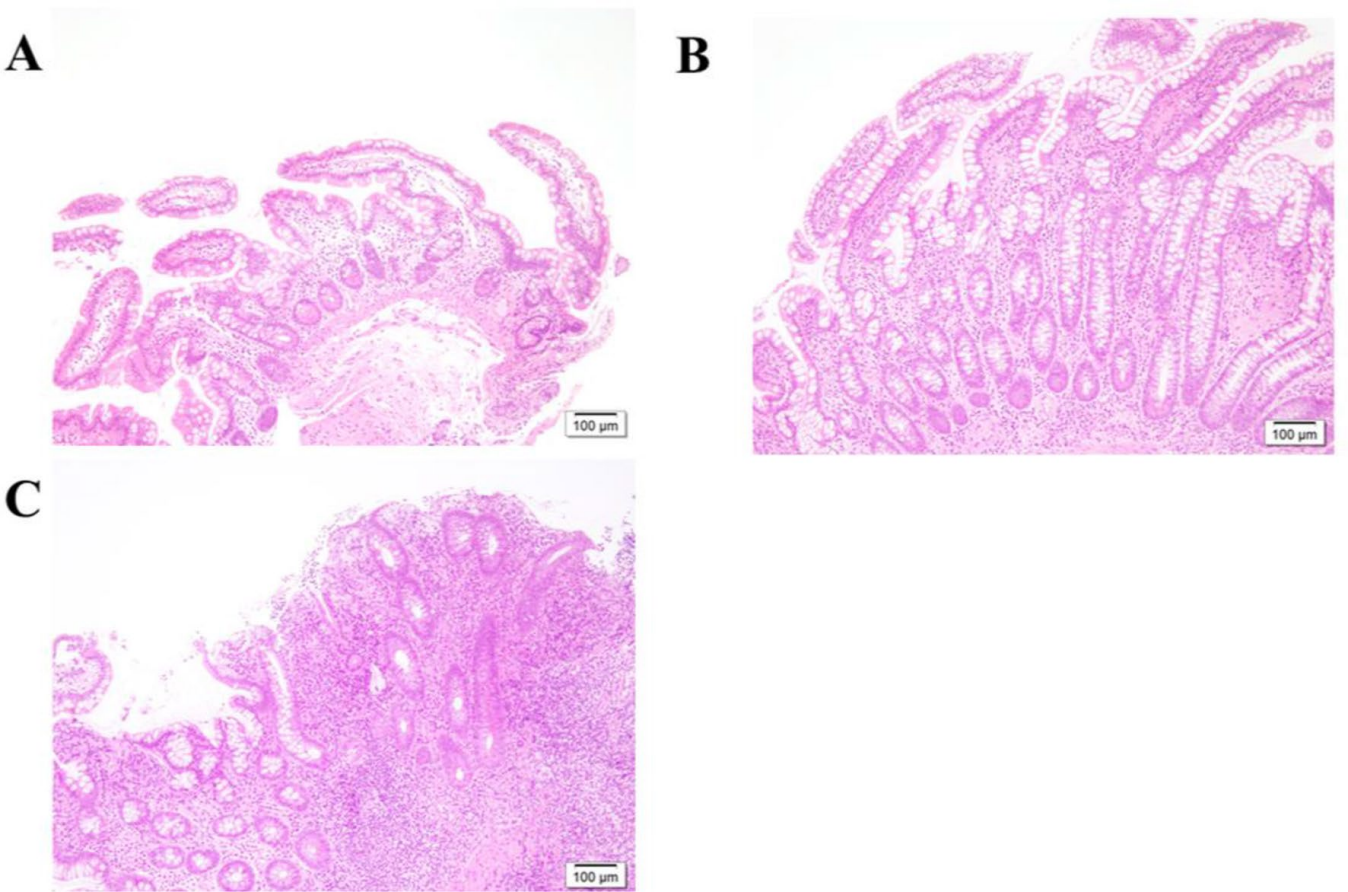
Typical cases of AL, PP and DP. 32 years-old, male, 1.3 years after the pouch usage. cPDAI = 0 (no symptoms). (**A**) AL, ePDAI = 0, hPDAI = 0, CMS = 1, SIBD = 2, Goblet cell rate = 14. (**B**) PP, ePDAI = 2, hPDAI = 2, CMS = 3, SIBD = 4, Goblet cell rate = 23. (**C**) DP, ePDAI = 2, hPDAI = 3, CMS = 4, SIBD = 4, Goblet cell rate = 35. *hPDAI-pouchitis disease activity index histology subscore; CMS-colonic metaplasia scores; SIBD-the IBD score.*

The morphological differences between AL, PP and DP are shown in Table 1. There were significant differences in all scores and findings between the pouch body and AL (*p* < 0.001), with the exception of Paneth cell metaplasia. The anal side showed a significantly higher hPDAI (AL/PP/DP; median score: 0/2/3), GC ratio (median ratio; 19%/24%/27%), and ePDAI (median score; 0/2/2) values in the order DP > PP > AL. The pouch body showed a significantly higher SIBD (median score; -2/1/1) and CMS (median score; 2/3/3) than the AL. Typical cases of AL, PP and DP are shown in Fig. 1.

**Table 1:** Differences between afferent limb and the pouch body.

Factors	AL	PP	DP	*p* Value		
				AL/PP	AL/DP	PP/DP
PDAI histology subscore	0 (0–1)	2 (1–3)	3 (1–4)	< 0.001	< 0.001	0.002
Polymorphic nuclear leukocyte infiltration (/1500um^2^) *	0 (0–0)	1 (0–2)	2 (0–4)	< 0.001	< 0.001	0.009
Crypt abscess	12 (4.6)	60 (23.0)	59 (22.6)	< 0.001	< 0.001	0.954
Normal (0) (0–1 neutrophil/1500um^2^)	225 (86.2)	123 (47.1)	101 (38.7)			
Mild (1) (2–3 neutrophil/1500um^2^)	18 (6.9)	56 (21.5)	57 (21.8)			
Moderate (2) (4–6 neutrophil/1500um^2^) + Crypt abscess	17 (6.5)	73 (28.0)	85 (32.6)	< 0.001	< 0.001	0.027
Severe (3) (7- neutrophil/500um^2^) + Crypt abscess	1 (0.4)	9 (3.4)	18 (6.9)			
Ulceration (per low-power field)*	0 (0–15)	15 (5–30)	20 (10–40)	< 0.001	< 0.001	0.017
0% (0)	134 (51.3)	67 (25.7)	46 (17.6)			
< 25% (1)	97 (37.2)	104 (39.8)	98 (37.5)			
25–50% (2)	20 (7.7)	49 (18.8)	63 (24.1)	< 0.001	< 0.001	0.009
> 50% (3)	10 (3.8)	41 (15.7)	54 (20.7)			
The IBD Score*	-2 (-2—-2)	1 (-2—3)	1 (-2—3)	< 0.001	< 0.001	0.162
Basal plasmacytosis with severe mononuclear infiltration	22 (8.7)	99 (37.9)	119 (45.6)	< 0.001	< 0.001	0.175
Crypt atrophy	10 (4.0)	52 (20.1)	63 (24.0)	< 0.001	< 0.001	0.527
Crypt distortion	43 (16.5)	120 (45.9)	119 (45.6)	< 0.001	< 0.001	0.953
Paneth cell metaplasia	261 (100.0)	261 (100.0)	261 (100.0)	N/A	N/A	N/A
Colonic metaplasia scores*	2 (1–2)	3 (2–4)	3 (3–4)	< 0.001	< 0.001	0.054
Villus length (um)*	346 (281–422)	280 (218–338)	259 (187–329)			0.034
Normal (385-um)	88 (33.7)	34 (13.0)	35 (13.4)			
Mild shortening (265-384um)	121 (46.4)	109 (41.8)	94 (36.0)	< 0.001	< 0.001	0.171
Moderate atrophy (176–264 um)	43 (16.5)	77 (29.5)	73 (28.0)			
Severe atrophy (−175 um)	9 (3.4)	41 (15.7)	59 (22.6)			
Crypt length (um)*	150 (127–170)	210 (173–225)	230 (184–284)	< 0.001	< 0.001	0.013
None (0) (−133 um)	79 (30.2)	15 (5.7)	10 (3.8)			
Mild (1) (134–190 um)	137 (52.5)	72 (27.6)	63 (24.1)	< 0.001	< 0.001	0.059
Moderate (2) (191–288 um)	37 (14.2)	126 (48.3)	125 (47.9)			
Extensive (3) (289 um)	8 (3.1)	48 (18.4)	63 (24.1)			
Goblet cell ratio (percent) *	19 (16–22)	24 (20–30)	27 (23–31)	< 0.001	< 0.001	< 0.001
PDAI endoscopy subscore*	0 (0–0)	2 (0–2)	2 (1–2)	< 0.001	< 0.001	0.026
Edema	9 (3.6)	24 (9.5)	29 (11.1)	0.003	< 0.001	0.562
Granularity	26 (10.0)	150 (57.3)	170 (65.1)	< 0.001	< 0.001	0.036
Friability	3 (1.2)	14 (5.5)	18 (7.1)	0.007	0.001	0.388
Loss of vascular pattern	27 (10.4)	164 (63.6)	188 (72.2)	< 0.001	< 0.001	0.018
Mucous exudate	5 (2.0)	17 (6.7)	21 (8.3)	0.004	0.001	0.512
Ulceration	4 (1.6)	23 (9.1)	22 (8.7)	< 0.001	< 0.001	0.889

Values in parentheses are percentages, unless indicated otherwise.

*Values are median (interquartile range).

*AL* afferent limbs; *PP* proximal pouch; *DP* distal pouch; *PDAI* Pouchitis disease activity index; *IBD* inflammatory bowel disease.

### Relationship between hPDAI, S_IBD_, CMS, GC ratio, ePDAI and the duration of pouch usage

Spearman’s correlation coefficients between the hPDAI, S_IBD_, CMS, GC ratio, ePDAI, and the duration of pouch usage (Duration) are shown in Table 2. The hPDAI was correlated with S_IBD_ (Spearman’s coefficient, r = 0.538; *p* < 0.001), CMS (r = 0.687; *p* < 0.001), and ePDAI (r = 0.552; *p* < 0.001), but not with the GC ratio (r = 0.175; *p* < 0.001) or Duration (r = −0.057; *p* = 0.107). The S_IBD_ was correlated with the CMS (r = 0.499; *p* < 0.001) and ePDAI (r = 0.487; *p* < 0.001), but not with the GC ratio (r = 0.257; *p* < 0.001) or Duration (r = 0.071; *p* = 0.046). The CMS was correlated with the ePDAI (r = 0.491; *p* < 0.001), but not correlated with the GC ratio (r = 0.262; *p* < 0.001) or Duration (r = 0.046; *p* = 0.196).

**Table 2:** Relationship between hPDAI S_IBD_, CMS, GC ratio, ePDAI and Duration.

	hPDAI	S_IBD_	CMS	GC ratio	ePDAI	Duration
hPDAI	1					
S_IBD_	0.538 (< 0.001)	1				
CMS	0.687 (< 0.001)	0.499 < 0.001	1			
GC ratio	0.175 (< 0.001)	0.257 (< 0.001)	0.262 (< 0.001)	1		
ePDAI	0.552 (< 0.001)	0.487 (< .001)	0.491 (< 0.001)	0.303 (< 0.001)	1	
Duration	- 0.057 (0.107)	0.071 (0.048)	0.046 (0.196)	0.050 (0.164)	0.039 (0.279)	1

Correlation between the scores and duration. Values shown are the Spearman correlation coefficients and *p* values.

*hPDAI* pouchitis disease activity index histology subscore; *S*_*IBD*_ the IBD Score; *CMS* colonic metaplasia scores; *GC ratio* goblet cell ratio; *ePDAI* pouchitis disease activity index endoscopy subscore; *Duration* duration of ileal pouch use.

### Relationship between histological inflammation, IBD-specific findings, and colonic metaplasia

The relationship between histological inflammation and IBD-specific findings is shown in Table 3. histological inflammation (hPDAI ≥ 3) was observed, especially in specimens with basal plasmacytosis with severe mononuclear cell infiltration (BP; odds ratio (OR) 6.790; 95% confidence interval (CI) 4.677–9.859; *p* < 0.001). In specimens with BP, we frequently found moderate or greater polymorphonuclear leukocyte infiltration (OR 6.027; 95% CI 4.156–8.740; *p* < 0.001; Supplementary Table S3A) and ulceration (OR 3.741; 95% CI 2.624–5.334; *p* < 0.001; Supplementary Table S3B).

**Table 3:** Relationship between histological inflammation (hPDAI ≥ 3) and the IBD-specific findings.

Variables	OR (95% CI)	*p*-value
Basal plasmacytosis with severe mononuclear cell infiltration	6.790 (4.677–9.859)	< 0.001
Crypt distortion	1.872 (1.292–2.714)	0.001
Crypt atrophy	3.301 (1.570–5.382)	< 0.001

*OR* odds ratio; *CI* confidence interval; hPDAI pouchitis disease activity index histology subscore; *IBD* inflammatory bowel disease.

**Figure 2:**
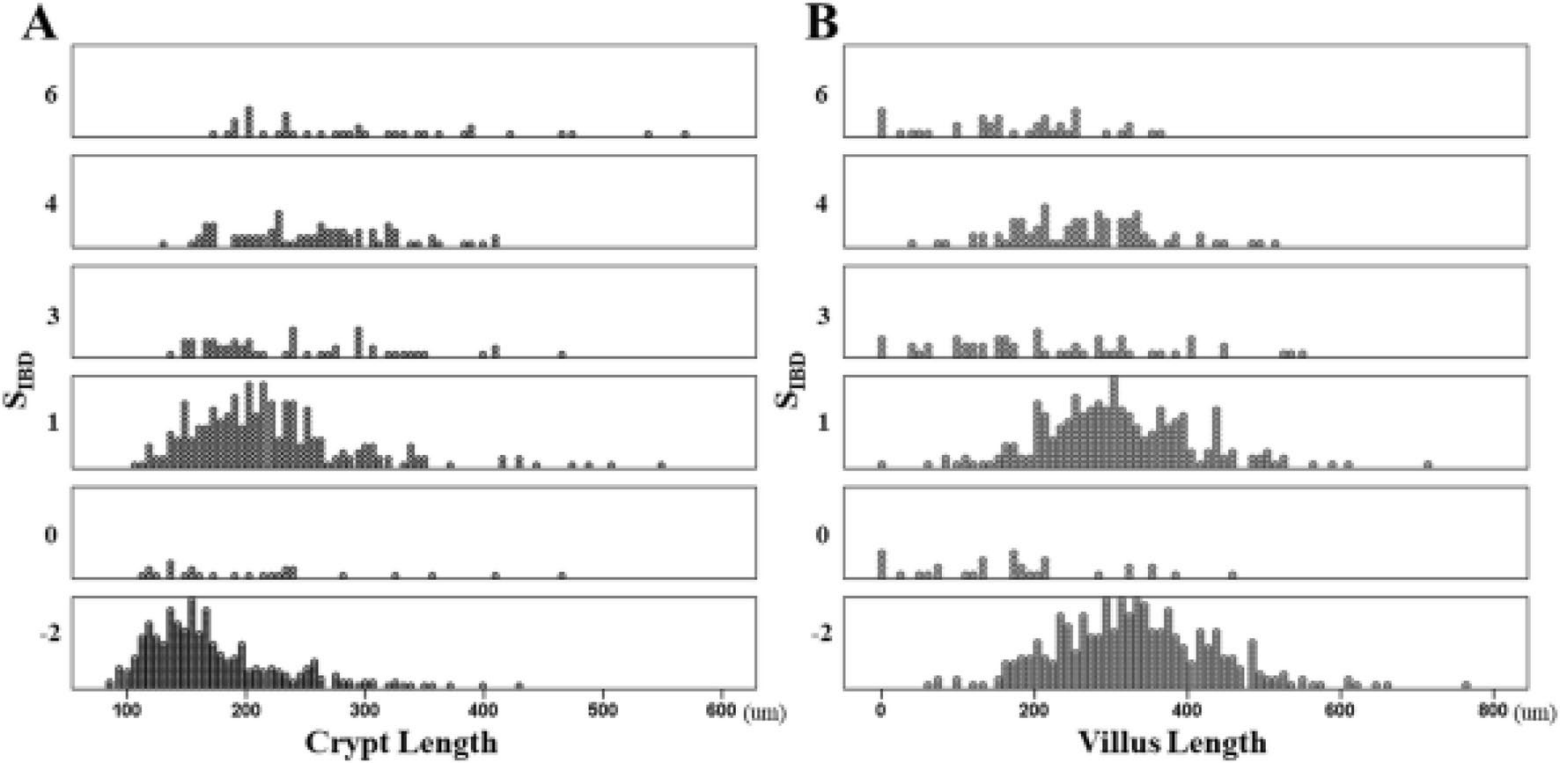
Correlation between CMS and S_IBD_. The crypt length correlated with the S_IBD_ [A; r = 0.505; *p* < 0.001], the villus length did not correlate with the S_IBD_ [B; r = −0.293; *p* < 0.001]. *CMS-colonic metaplasia scores; SIBD-the IBD score.*

The relationship between IBD-specific findings and colonic metaplasia is shown in Fig. 2. The crypt length was correlated with the S_IBD_ (r = 0.505; *p* < 0.001), but the villus length was not (r = −0.293; *p* < 0.001). In specimens with moderate or greater crypt hyperplasia, we frequently found BP (OR 3.414; 95% CI 2.425–4.805; *p* < 0.001; Supplementary Table S4A, crypt atrophy (OR 5.248; 95% CI 3.397–8.108; *p* < 0.001; Supplementary Table S4B, and crypt distortion (OR 4.886; 95% CI 3.513–6.797; *p* < 0.001; Supplementary Table S4C).

**Figure 3:**
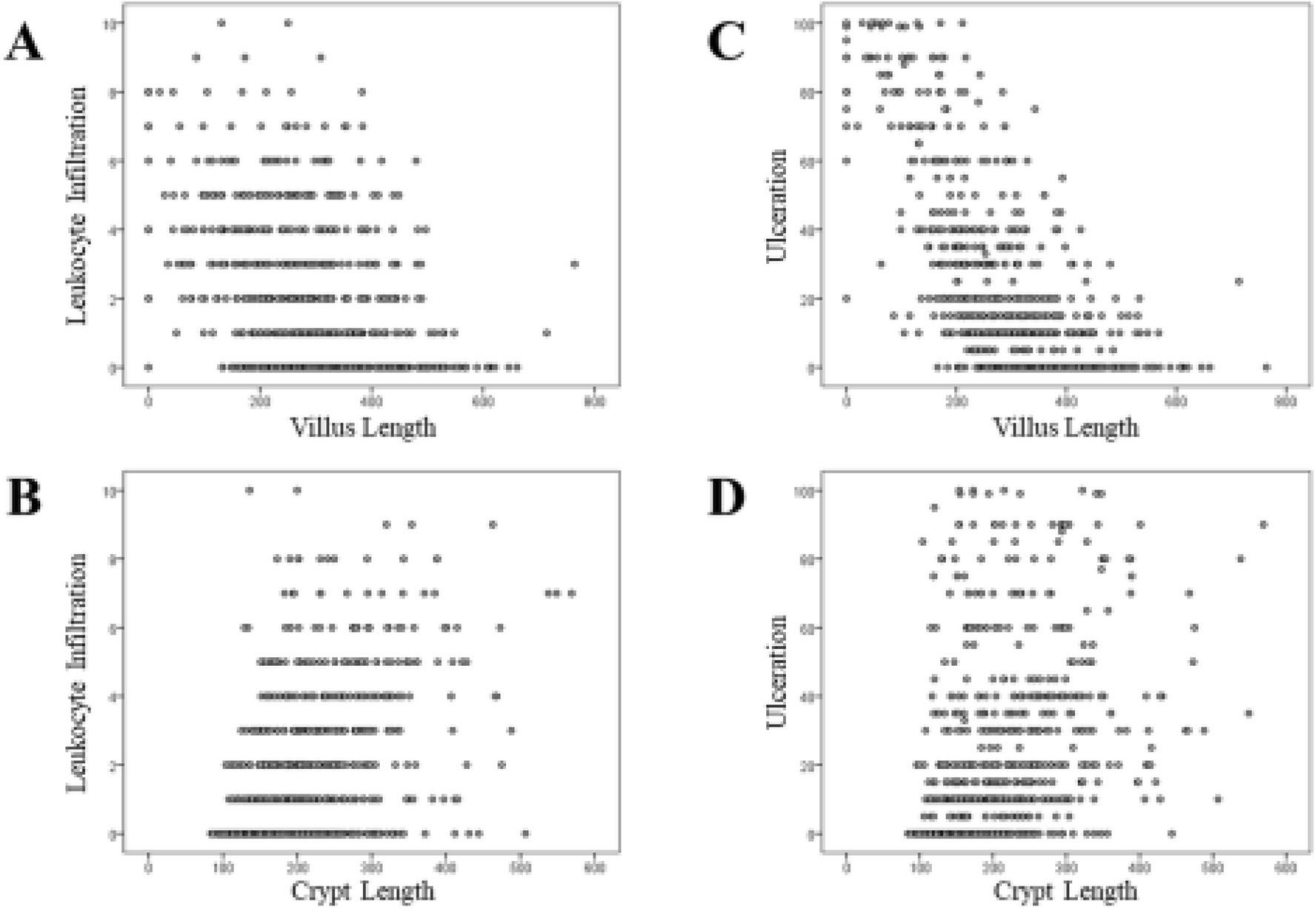
Correlation between findings of hPDAI and CMS. Neutrophil infiltration did not correlated with villus length [**A**; Spearman’s coefficient r = −0.367; *p* < 0.001], but did correlated with crypt length [**B**; r = 0.469; *p* < 0.001]. Ulceration showed an inverse correlation with villi length [**C**; r = −0.659; *p* < 0.001], but not with crypt length [**D**; r = 0.362; *p* < 0.001]. *hPDAI-pouchitis disease activity index histology subscore; CMS-colonic metaplasia scores.*

The relationship between colonic metaplasia and histological inflammation is shown in Fig. 3. The number of infiltrating neutrophils in the lamina propria was correlated with the crypt length (r = 0.442; *p* < 0.001) but not with villus length (r = −0.367; *p* < 0.001). The ulceration range showed an inverse correlation with the villus length (r = −0.659; *p* < 0.001) but not with the crypt length (r = 0.362; *p* < 0.001).

## Discussion

We evaluated a total 142 UC patients, including 261 pouchoscopies. There were significant differences between the pouch body and AL in histological inflammation, IBD-specific findings, colonic metaplasia, the GC ratio, and endoscopic inflammation.

Histological inflammation was more common in the pouch body than in the AL, similar to previous reports^10,11^. Various hypotheses regarding the etiology of inflammation of the UC pouch have been proposed, including dysbiosis of the ileal pouch microbiota, deprivation of nutritional short chain fatty acids (SCFAs), recurrence of IBD, mucosal ischemia, genetic predisposition, immune dysregulation^12^, autophagy^13^ and apotosis^14^.

The main differences between the pouch body and the AL may be changes in the microbiota and SCFAs due to fecal stasis. These changes may contribute to pouchitis in many patients, since most cases of pouchitis improve with antibiotics. The increased abundance of *Clostridium perfringens*^15^, *Enterobacteriaceae*^16^, *Escherichia coli*^16^, *Fusobacterium spp*^17^, *Ruminococcus obeum*^17^ has been shown to be associated with pouchitis. Butyrate, a metabolite of butyric acid-producing bacteria, promotes colonic health by reducing oxidative stress, acting as an anti-inflammatory factor^18^. A reduced butyrate level may therefore result in damage to the intestinal epithelial cell barrier^19^. These studies suggest that changes in the microbiota and SCFAs are involved in the development of pouchitis. However, it is difficult to explain how such changes themselves are associated with the increased incidence of pouchitis in UC patients.

Regarding genetic predispositions and immune dysregulation, the NOD2 variant carries an inherent risk of pouchitis and the function of the Nod2 gene is to recognize bacteria, and to stimulate the enteric immune system^20^. Regarding autophagy and apoptosis, the UC pouch without inflammation shows modulation of autophagy markers^13^ and high levels of pro-apoptotic activity, even in the pouch^14^. These findings suggest that an unidentified IBD pathogenesis is involved in the inflammation in the UC pouch.

We newly found that IBD-specific findings are more common in the pouch body than in the AL. Since pouchitis is considerably more common in the patients with UC than in those with FAP (22–50% vs. 0–11%)^6,21^, pouchitis may represent the recurrence of underlying IBD within the neorectum^12,22^. The IBD-specific findings we reviewed here are those that occur specifically in colonic tissue with IBD, and are consistent with findings in the small intestinal tissue with chronic inflammation in Crohn's disease^23^. Histological inflammation was strongly associated with BP in this study. BP is an IBD-specific finding that may be involved in the pathogenesis of IBD^8,24^. It preceds acute inflammation in non-inflamed areas of UC^25^ and is also a consensus indicator of UC activity^26^. Thus, inflammation in the pouch with BP may be IBD-specific pouchitis. Although the etiology of IBD-specific findings is not yet known, IBD-specific findings occur more frequently in specimens with crypt hyperplasia. An increase in crypt length is described as hyperplasia, while a decrease in crypt density is described as atrophy. Since these two changes occur simultaneously, “crypt extension” may be an appropriate term to describe such changes in the crypt morphology. Although the histogenesis of crypt distortion and BP in UC is unclear^27^, crypt extension may be associated with crypt distortion and BP, as crypt distortion and BP were more frequently observed in cases with a longer crypt length.

It is often thought that colonic metaplasia, including villus atrophy and crypt hyperplasia, is a consequence of the subsequent inflammation. A previous report demonstrated that colonic metaplasia is associated with histological inflammation and is not the result of long-term adaptation^9^. Supporting the finding that colonic metaplasia is related to inflammation independently of the time course, inflammation diminished with time, and colonic metaplasia also diminished in some cases (Supplementary Fig. S1). Ulceration was inversely correlated with villus length, and the number of infiltrating neutrophils was correlated with crypt length. Villus atrophy is caused by epithelial cell damage, as occurs in celiac disease^28^. The increase in the number of neutrophils and other inflammatory cells in the lamina propria may increase the cell density in the lamina propria, and the thickening of the lamina propria associated with this increase may result in crypt hyperplasia. Colonic metaplasia including crypt hyperplasia and villus atrophy seems to be a non-IBD specific finding, as it is also seen in the intestinal mucosa of celiac disease in Marsh type 3^29^ and in the ileal pouch of normal mice^30^.

An increased GC ratio is also a non-IBD-specific findings, as it is also seen in normal mice with IPAA^31^. Specific commensal bacteria and SCFA are important in GC differentiation^32^. Changes in the microbiota and SCFAs due to fecal stasis may increase the proportion of GCs especially in pouch body. In the present study, the GC ratio was not correlated with endoscopic, histological inflammation, or IBD-specific findings, despite the fact that the deletion of GCs is a common feature in the colonic mucosa of UC^33^. Changes in the GC ratio may have little effect on pouchitis or the recurrence of IBD.

Histological inflammation was found to be associated with the endoscopic inflammation. It is widely believed that the clinical symptoms of pouchitis are not correlated with endoscopic or histological inflammation due to the presence of cuffitis and irritable pouch syndrome (IPS)^11,34^. As in previous studies, PDAI clinical subscore (cPDAI) was not correlated with the ePDAI (r = 0.151, *p* < 0.001), or the hPDAI (r = 0.189, *p* < 0.001) in our study (Supplementary Fig. S2). Although the correlation between histological and endoscopic inflammation remains unclear^11,35^, it is natural to assume that histological and endoscopic inflammation are correlated, since the modified PDAI (mPDAI), which omits the histological assessment, can predict the PDAI scores with high accuracy^36^. A total of 24% patients who were negative for mPDAI but positive for PDAI had symptoms of pouchitis (Supplementary Fig. S3). A histological assessment may be helpful when patients are suffering from the symptoms of pouchitis. Pouchitis should be considered when friability, ulceration, or mucous exudate are observed, as histological inflammation (hPDAI ≥ 3) exists in > 75% of patients with these findings (Supplementary Fig. S4).

The present study was associated with three limitations. First, the study included only patients with UC. No report is available on the utility of the IBD score in the small intestine, and it has not been proven that the IBD score is truly an IBD-specific finding even in the small intestine. We are planning a large multicenter study including non-IBD (FAP) patients. Second, over 75% of the patients were asymptomatic (cPDAI = 0). However, the data from this study are valuable because they reflect the whole view of the ileal pouch, since almost all patients with an ileal pouch were regularly evaluated by follow-up pouchoscopy. Third, the histological assessment was not blinded. Therefore, subjective evaluation findings (e.g., the degree of polymorphonuclear leukocyte and mononuclear cell infiltration, villus atrophy, and crypt hyperplasia). were objectively evaluated by measuring the number or length.

The strength of the study is the detailed evaluation of a large sample of 261 endoscopes and 783 biopsy tissues. As a result, we found not only morphological difference between the pouch and AL, but also the correlation between the findings.

In conclusion, (1) histological inflammation, (2) IBD-specific findings, (3) colonic metaplasia, (4) the GC ratio, and (5) endoscopic inflammation were increased in the pouch body in comparison to the AL in patients with UC. Histological inflammation, IBD-specific findings, and colonic metaplasia, occur together and correlate with endoscopic inflammation in the ileal pouch.

## Methods

### Study population and material

**Figure 4:**
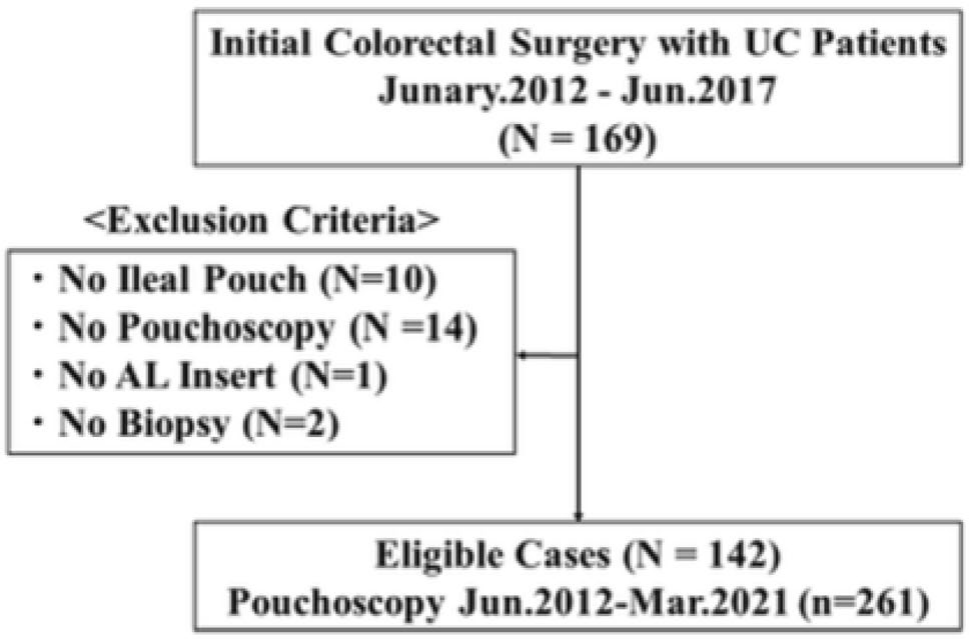
Outline of patient selection. *UC-Ulcerative colitis; AL-afferent limbs.*

The study population and materials used are shown in Fig. 4. From January 2012 to June 2017, 169 consecutive patients who underwent initial surgical resection for UC were enrolled from Yokohama City University Medical Center in Japan. The exclusion criteria were as follows: (1) no ileal pouch construction, (2) no pouchoscopy, (3) no biopsy of the AL or the pouch body.

The surgical indications, strategy and procedure are as previously reported^37,38^. In brief, for patients whose surgical indications were severe or refractory colitis, stapled ileal pouch-anal anastomosis (IPAA) was performed. Single-stage surgery under hand-assisted laparoscopic proctocolectomy was performed when standby surgery was possible. The other patients received a modified two-stage surgery. For patients whose surgical indication was cancer or dysplasia, hand-assisted laparoscopic proctocolectomy with hand-sewn anastomosis and diverting ileostomy was performed (the ileostomy closed at 12 weeks after the initial surgery). A 12-cm-long ileal J-pouch was constructed in all patients. The first pouchoscopy was performed at one year after pouch construction, and every two years thereafter as routine clinical practice. Pouchitis was diagnosed by the clinician based on symptoms and the endoscopic findings, and an antibiotic was prescribed.

A total of 142 patients and 261 pouchoscopies (until March 2021) were enrolled. The characteristics of the study population are shown in Supplementary Tables S1 and S2. Pouchitis was experienced in 29 cases (20%) during the follow-up period, and over 75% of patients were asymptomatic at the time of endoscopy. No cases of secondary pouchitis diagnosed as ischemia or with surgical complications were observed in this study.

### Endoscopic and clinical evaluation

Segmental evaluation of the anal transitional zone (rectal cuff), pouch body (including PP and DP), and AL were performed. The rectal cuff was defined as the area from the dentate line to the anal ileal-pouch anastomosis, DP was defined as the anastomotic site to the anal verge (AV) 10 cm, PP was defined as AV 10 cm to AV 16 cm, and AL was defined as AV 16 cm to 22 cm. Endoscopic inflammation was assessed using ePDAI^7^. Each endoscopic finding was diagnosed by two gastrointestinal surgeons in the IBD center.

Clinical evaluations were collected using clinical report forms. All patients were surveyed for stool frequency, medication status and clinical symptoms according to the PDAI clinical subscore (cPDAI) at the time of pouchoscopy^7^.

### Histopathological evaluation

Biopsies were taken from the most endoscopically inflamed sites in each segment (AL, PP, DP, and the rectal cuff). Hematoxylin and eosin-stained histological sections from biopsy specimens of the AL, PP, and DP were reexamined. Biopsy specimens of the rectal cuff were not reexamined. We evaluated histological inflammation, IBD-specific findings, colonic metaplasia, and the GC ratio for each specimen. Histological inflammation was assessed using hPDAI^7^. IBD-specific findings were assessed using the IBD Score (S_IBD_)^8^. The definition of Paneth cell metaplasia in this study was assessed for the presence of Paneth cells in each specimen in order to be closer to the method originally used^8^. Since Paneth cells are resided in the small intestine, Paneth cell metaplasia in the pouch may not be pathologically appropriate (If Paneth cells reappeared after the pouch was transformed into colonic tissue by colonic metaplasia, it may be appropriate.), and we believe that sufficient caution should be exercised in using the term "Paneth cell metaplasia" in the pouch. Colonic metaplasia was assessed using CMS^9^. The GC ratio was calculated as the average ratio of GCs to epithelial cells in the villus. Findings that required a subjective evaluation were objectively assessed by measuring the number or length, and cutoff values were determined (polymorphic nuclear leukocyte infiltration in the hPDAI as the number of neutrophils in the lamina propria, severe mononuclear cell infiltration in the S_IBD_ as the number of mononuclear cells in the lamina propria, and villus atrophy and crypt hyperplasia in the CMS as the average length of the villus and crypt).

### Statistical analyses

Descriptive statistics were reported as frequencies and percentiles for categorical variables and the median and interquartile range for continuous variables. Differences between categorical variables were tested using chi-squared test, and continuous variables were tested using the Mann–Whitney *U* test. The relationship between categorical variables were tested using a logistic regression analysis, while those between continuous variables were tested using Spearman’s correlation coefficient. *p*-values of < 0.05 were considered statistically significant. The Bonferroni method was used when multiple comparisons were conducted.

Statistical analyses were performed using the SPSS software program (version 22.0, SPSS Inc., Chicago, IL, USA).

### Ethical considerations

This study was performed in accordance with the Declaration of Helsinki and study protocol was approved by the Ethical Advisory Committee of Yokohama City University Graduate School of Medicine (Registration no. B210400067). Informed consent was obtained from all participants.

## Supplementary Information

Supplementary Information.

## Data Availability

The data underlying this article cannot be shared publicly per the Yokohama City University Medical Center’s Institutional Review Board policy to preserve the privacy of individuals that participated in the study. The data will be shared on reasonable request to the corresponding author.
