# Supplementary material for: Inflammatory bowel disease-specific findings are common morphological changes in the ileal pouch with ulcerative colitis
Source: Sci Rep. 2022 Nov 27;12:20361. doi: 10.1038/s41598-022-24708-2 (PMC9701810; doi:10.1038/s41598-022-24708-2)
Supplement: Supplementary file 1 — Supplementary Information. [file 41598_2022_24708_MOESM1_ESM.docx]

| **Variables** | **Patients**  **(N=142)** | |
| --- | --- | --- |
| Gender  Male  Female | 94  48 | (66.2)  (33.8) |
| Age at pouch surgery　(years)* | 37 | (24-52) |
| Time elapsed since UC onset　(years)* | 4.5 | (3.0-12.0) |
| Age at UC onset to initial surgery (years)* | 27 | (18-39) |
| Disease extent  Extensive colitis  Left-sided colitis  Proctitis | 139  3  0 | (97.9)  (2.1)  (0) |
| Surgical indication  Severe colitis  Refractory colitis  Dysplasia/ CAC | 39  92  11 | (27.5)  (64.8)  (7.7) |
| Staged surgery  Single-Staged surgery  Modified two-staged surgery | 69  73 | (48.6)  (51.4) |
| Type of anastomosis  Hand-sewn IPAA  Stapled IPAA | 9  133 | (6.3)  (93.7) |
| Rectal remnant length (cm)* | 2.0 | (1.5-2.5) |
| Anastomosis leakage | 11 | (7.7) |
| Extra-intestinal manifestations | 23 | (16.2) |
| Auto-immune disease | 21 | (14.7) |
| Postoperative follow-up period (year)* | 4.7 | (4.4-5.1) |
| Number of pouchoscopy | 2 | (1-3) |
| Pouchitis | 29 | (20) |
| Postoperative oncogenesis | 0 | (0) |
| Pouch failure | 0 | (0) |

**Supplementary Table S1. Characteristics of study population (patients)**

Values in parentheses are percentages, unless indicated otherwise

*Values are median (interquartile range)

*CAC-Colitis associated cancer; UC-Ulcerative Colitis; IPAA-ileal pouch-anal anastomosis.*

| **Variables** | **Pouchoscopy**  **n=261** | |
| --- | --- | --- |
| Gender  Male  Female | 175  86 | (67)  (33) |
| Age at pouchoscopy (years)* | 39 | (26-51) |
| Duration of ileal pouch use (years)* | 1.7 | (1.2-3.6) |
| Stool frequency (per day) * | 7 | (5-10) |
| PDAI clinical subscore*  Increased stool frequency  Rectal bleeding  Fecal urgency or abdominal cramps  Fever | 0  21  21  14  1 | (0-0)  (8)  (8)  (5)  (0) |
| Use of Antibiotic  Use of Probiotics  Use of Berberine chloride  Use of loperamide  Use of Steroid  Use of IBD drugs  Use of NSAIDs  Use of PPI or H2 broker | 10  197  179  65  7  2  2  40 | (4)  (75)  (69)  (25)  (3)  (1)  (1)  (15) |

**Supplementary Table S2. Characteristics of study population (pouchoscopy)**

Values in parentheses are percentages, unless indicated otherwise

*Values are median (interquartile range)

*PDAI-Pouchitis disease activity index; IBD drugs-Inflammatory bowel disease drugs including steroids, amino salicylates, immunomodulators and biologics; NSAIDs-Nonsteroidal anti-inflammatory drugs; PPI-Proton pump inhibitor; H2 broker-Histamine H2-receptor antagonist.*

**Supplementary Table S3A. Relationship between** **polymorphic nuclear leukocyte infiltration (4** **neutrophil/ 1500um^2^-) and the IBD-specific findings**

| **Variables** | **OR (95% CI)** | **P-value** |
| --- | --- | --- |
| Basal plasmacytosis with severe mononuclear cell infiltration | 6.027 (4.156-8.740) | < 0.001 |
| Crypt distortion | 2.191 (1.510-3.178) | < 0.001 |
| Crypt atrophy | 3.510 (2.574-5.377) | < 0.001 |

*OR-odds ratio; CI-confidence interval; IBD-inflammatory bowel disease.*

**Supplementary Table S3B. Relationship between ulceration (25%-) and the IBD-specific findings**

| **Variables** | **OR (95% CI)** | **p-value** |
| --- | --- | --- |
| Basal plasmacytosis with severe mononuclear cell infiltration | 3.741 (2.624-5.334) | < 0.001 |
| Crypt distortion | 1.376 (0.966-1.958) | 0.077 |
| Crypt atrophy | 2.671 (1.901-3.590) | < 0.001 |

*OR-odds ratio; CI-confidence interval; IBD-inflammatory bowel disease*

**Supplementary Table S4A. Relationship between basal plasmacytosis with severe mononuclear cell infiltration and colonic metaplasia scores**

| **Variables** | **OR (95% CI)** | **p-value** |
| --- | --- | --- |
| Villus atrophy (villus length<264um) | 2.814 (2.032-3.897) | < 0.001 |
| Crypt hyperplasia (crypt length>191um) | 3.414 (2.425-4.805) | < 0.001 |

*OR-odds ratio; CI-confidence interval.*

**Supplementary Table S4B. Relationship between crypt atrophy and colonic metaplasia scores**

| **Variables** | **OR (95% CI)** | **p-value** |
| --- | --- | --- |
| Villus atrophy (villus length<264um) | 2.429 (1.560-3.783) | < 0.001 |
| Crypt hyperplasia (crypt length>191um) | 5.248 (3.397-8.108) | < 0.001 |

*OR-odds ratio; CI-confidence interval.*

**Supplementary table S4C. Relationship between crypt distortion and colonic metaplasia scores**

| **Variables** | **OR (95% CI)** | **p-value** |
| --- | --- | --- |
| Villus atrophy (villus length<264um) | 0.965 (0.700-1.331) | 0.830 |
| Crypt hyperplasia (crypt length>191um) | 4.886 (3.513-6.797) | < 0.001 |

*OR-odds ratio; CI-confidence interval.*

**Supplementary Figure S1. Morphological changes with duration**


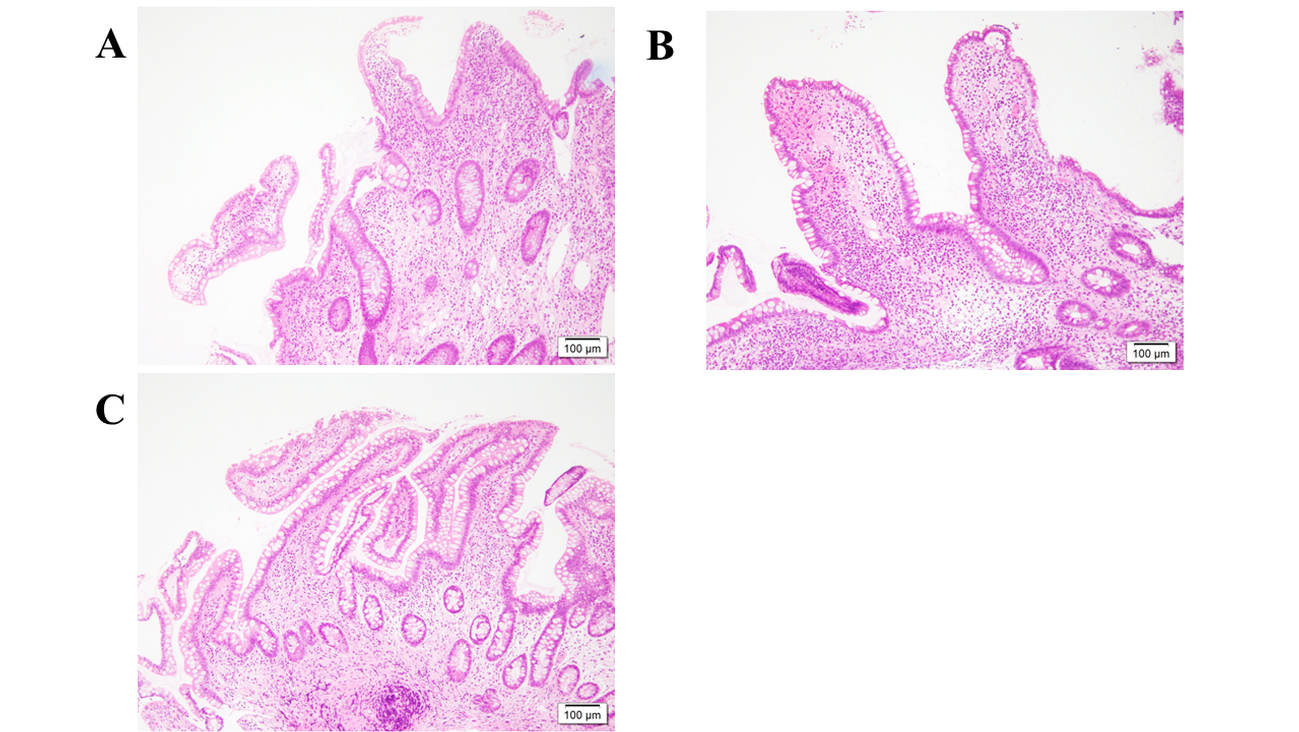


Longitudinal biopsy specimens of pouchoscopy. 33 years-old, male at the initial operation. (A) 1 year after the pouch use. hPDAI=4, CMS=3, SIBD=3. (B) 3 years after the pouch use. hPDAI=2, CMS=3, SIBD=4. (C) 5 years after the pouch use. hPDAI=0, CMS=2, SIBD=0.

*hPDAI-pouchitis disease activity index histology subscore; CMS-colonic metaplasia scores; SIBD-the IBD score.*

**Supplemental Figure S2. Relationship between PDAI subscores**


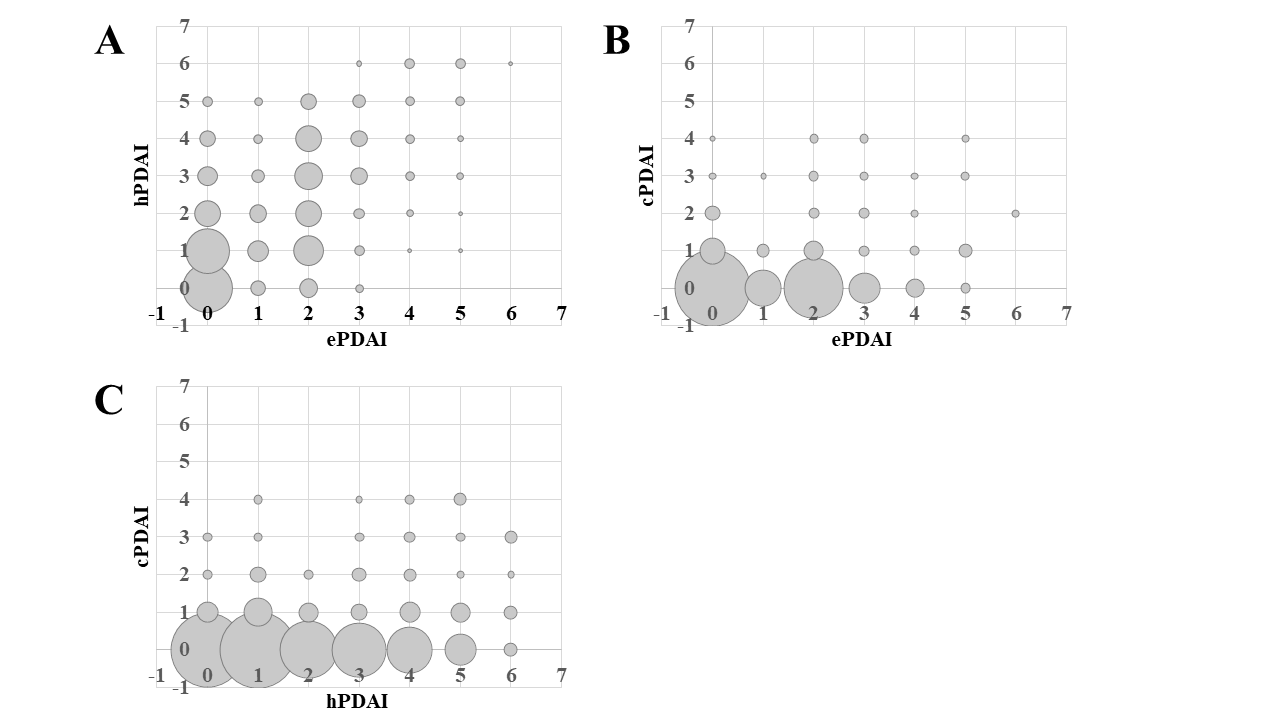


Correlation between PDAI subscores. (A) hPDAI was correlated to ePDAI (Spearman correlation coefficients r=0.552, p<0.001), (B) cPDAI was not correlated to ePDAI (r=0.151, p<0.001), (C) cPDAI was not correlated to hPDAI (r=0.189, p<0.001).

*PDAI-pouchitis disease activity index; hPDAI-PDAI histology subscore; ePDAI-PDAI endoscopy subscore; cPDAI-PDAI clinical subscore.*

**Supplemental Figure S3. Relationship between ePDAI and hPDAI**


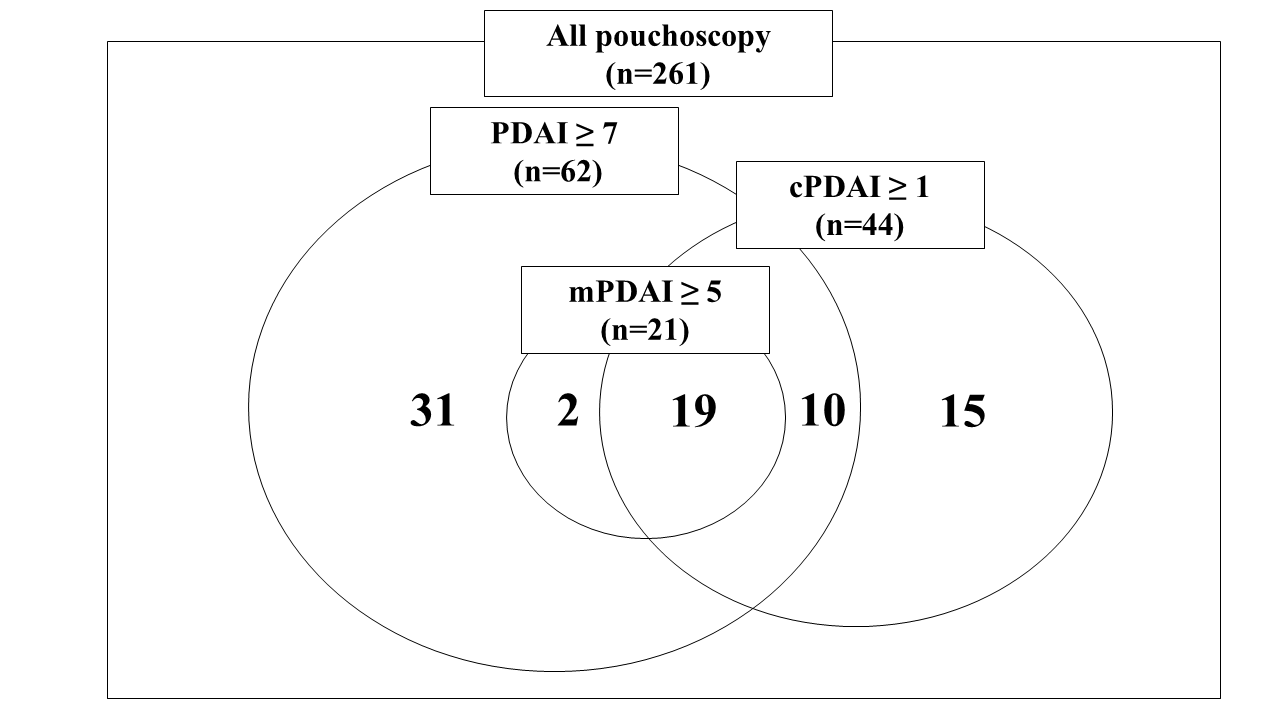


10 of the 41 (24%) patients who were negative for mPDAI, but positive for PDAI had symptoms of pouchitis.

*PDAI-pouchitis disease activity index; cPDAI-PDAI clinical subscore.*

**Supplemental Figure S4. Relationship between ePDAI and hPDAI**


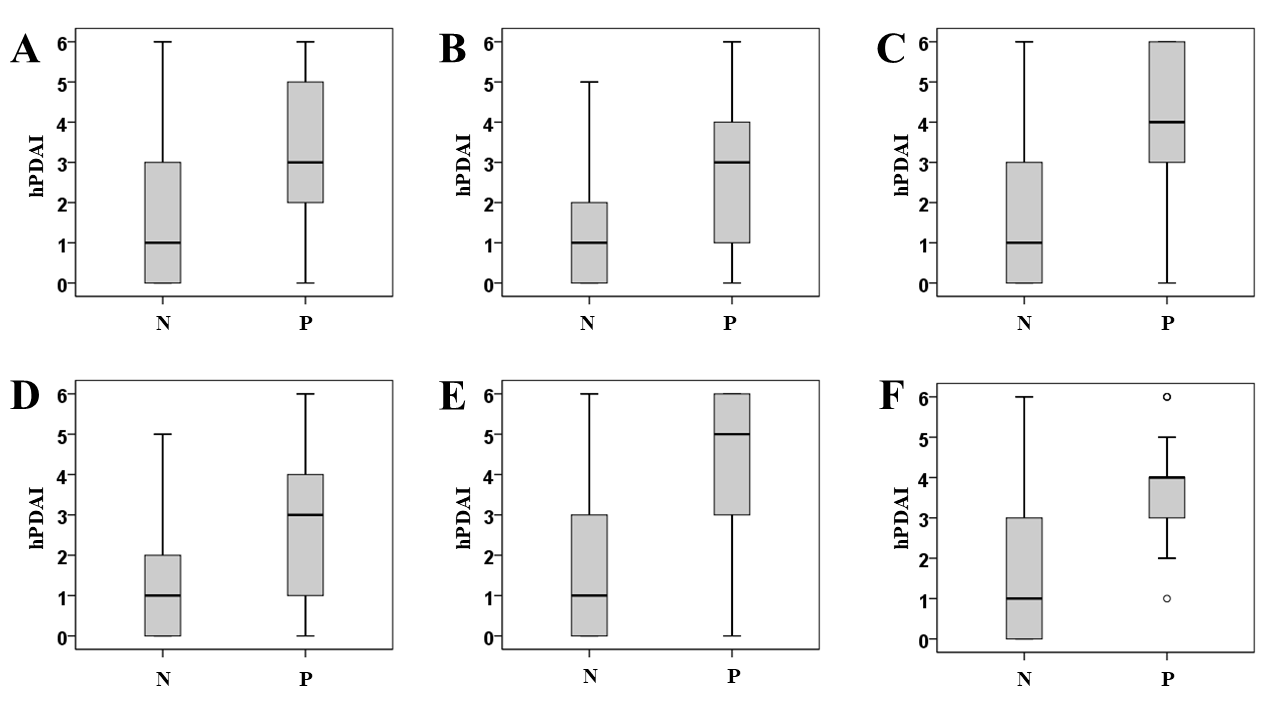


Histological inflammation (hPDAI≥3) is present in over 75% of patients with friability, ulceration or mucous exudate. (A) Edema, (B) Granularity, (C) Friability, (D) Loss of Vascular Pattern, (E) Mucous Exudate, (F) Ulceration.

*PDAI-pouchitis disease activity index; ePDAI-PDAI endoscopy subscore; hPDAI-PDAI histology subscore; N-negative; P-positive.*
